# Trial Sponsorship and Time to Reporting for Phase 3 Randomized Cancer Clinical Trials

**DOI:** 10.3390/cancers12092636

**Published:** 2020-09-16

**Authors:** Timothy A. Lin, Clifton David Fuller, Vivek Verma, Walker Mainwaring, Andres F. Espinoza, Austin B. Miller, Amit Jethanandani, Dario Pasalic, Prajnan Das, Bruce D. Minsky, Charles R. Thomas, David R. Fogelman, Vivek Subbiah, Ishwaria M. Subbiah, Ethan B. Ludmir

**Affiliations:** 1 Department of Radiation Oncology, The University of Texas MD Anderson Cancer Center, 1515 Holcombe Blvd, Houston, TX 77030, USA; tlin50@jh.edu (T.A.L.); cdfuller@mdanderson.org (C.D.F.); vivek333@gmail.com (V.V.); DPasalic@mdanderson.org (D.P.); PrajDas@mdanderson.org (P.D.); bminsky@mdanderson.org (B.D.M.); 2 Department of Radiation Oncology and Molecular Sciences, Sidney Kimmel Comprehensive Cancer Center, Johns Hopkins University School of Medicine, 401 N. Broadway Baltimore, MD 21287, USA; 3 Lankenau Medical Center, 100 E Lancaster Ave, Wynnewood, PA 19096, USA; mainwaringw@mlhs.org; 4 Baylor College of Medicine, 1 Baylor Plaza, Houston, TX 77030, USA; Andres.Espinoza@bcm.edu; 5 McGovern Medical School, The University of Texas Health Science Center, 7000 Fannin, Suite 1880, Houston, TX 77030, USA; austinmiller@llu.edu; 6 Department of Radiation Oncology, The University of Miami Sylvester Comprehensive Cancer Center, 1475 NW 12th Ave, Miami, FL 33136, USA; amit.jethanandani@jhsmiami.org; 7 Department of Radiation Medicine, Oregon Health and Science University, 3181 SW Sam Jackson Park Rd, Portland, OR 97239, USA; thomasch@ohsu.edu; 8 Department of Gastrointestinal Medical Oncology, The University of Texas MD Anderson Cancer Center, 1515 Holcombe Blvd, Houston, TX 77030, USA; dfogelman@mdanderson.org; 9 Department of Investigational Cancer Therapeutics, The University of Texas MD Anderson Cancer Center, 1515 Holcombe Blvd, Houston, TX 77030, USA; VSubbiah@mdanderson.org; 10 Department of Palliative, Rehabilitational, and Integrative Medicine, The University of Texas MD Anderson Cancer Center, 1515 Holcombe Blvd, Houston, TX 77030, USA; ISubbiah@mdanderson.org

**Keywords:** clinical trials, industry funding, cooperative groups, health policy

## Abstract

**Simple Summary:**

Timely execution and reporting of cancer clinical trials is important to patients and clinical researchers. Looking at a group of over three hundred cancer clinical trials with published results, we studied what factors influenced how quickly they reported their results in scientific journals. We found that trials sponsored by cooperative groups tended to report more slowly on average, while industry-funded trials reported more quickly. Additionally, we found that even after accounting for other variables that might affect timely reporting—such as whether a trial was successful, the type of cancer studied, or what type of intervention was studied—industry-funded trials still reported more quickly on average than non-industry funded trials. The reasons for these differences in reporting times are important to understand because trial results affect patient care and ongoing clinical research endeavors.

**Abstract:**

The pace of clinical trial data generation and publication is an area of interest within clinical oncology; however, little is known about the dynamics and covariates of time to reporting (TTR) of trial results. To assess these, ClinicalTrials.gov was queried for phase three clinical trials for patients with metastatic solid tumors, and the factors associated with TTR from enrollment completion to publication were analyzed. Based on the 319 included trials, cooperative-group-sponsored trials were reported at a slower rate than non-cooperative-group trials (median 37.5 vs. 31.0 months; *p* < 0.001), while industry-funded studies were reported at a faster rate than non-industry-supported trials (31.0 vs. 40.0 months; *p* = 0.005). Furthermore, successful trials (those meeting their primary endpoint) were reported at a faster rate than unsuccessful studies (27.5 vs. 36.0 months; *p* < 0.001). Multivariable analysis confirmed that industry funding was independently associated with a shorter TTR (*p* = 0.006), while cooperative group sponsorship was not associated with a statistically significant difference in TTR (*p* = 0.18). These data underscore an opportunity to improve cooperative group trial efficiency by reducing TTR.

## 1. Introduction

The pace of evidence generation from clinical trials is an area of active interest within clinical oncology [1]. Phase three clinical trials in oncology are time-intensive undertakings with numerous regulatory steps, requiring coordination amongst several stakeholders [2]. A report published by the Institute of Medicine in 2010 highlighted the need for the consolidation and streamlining of trial-related processes to reduce the time spent on clinical trial conception, initiation, and accrual [3]. Complicating these efforts is a decline in government support for clinical trials in recent years. This has subsequently led to a greater reliance on industry support, particularly for larger late-phase trials [4]. Efforts made to understand delays in clinical trial activation and to reduce the time taken from trial conception to the launch of cooperative group trials appear promising [2,5,6,7]. Further studies have focused on understanding and reducing delays in the clinical trial accrual process [8]. However, less is known about the factors associated with time to reporting (TTR) following the completion of accrual. To that end, we sought to identify the factors associated with the speed of post-accrual reporting. We hypothesized that industry funding and cooperative-group-sponsorship may be associated with shorter and longer TTR, respectively. 

## 2. Results

**Figure 1:**
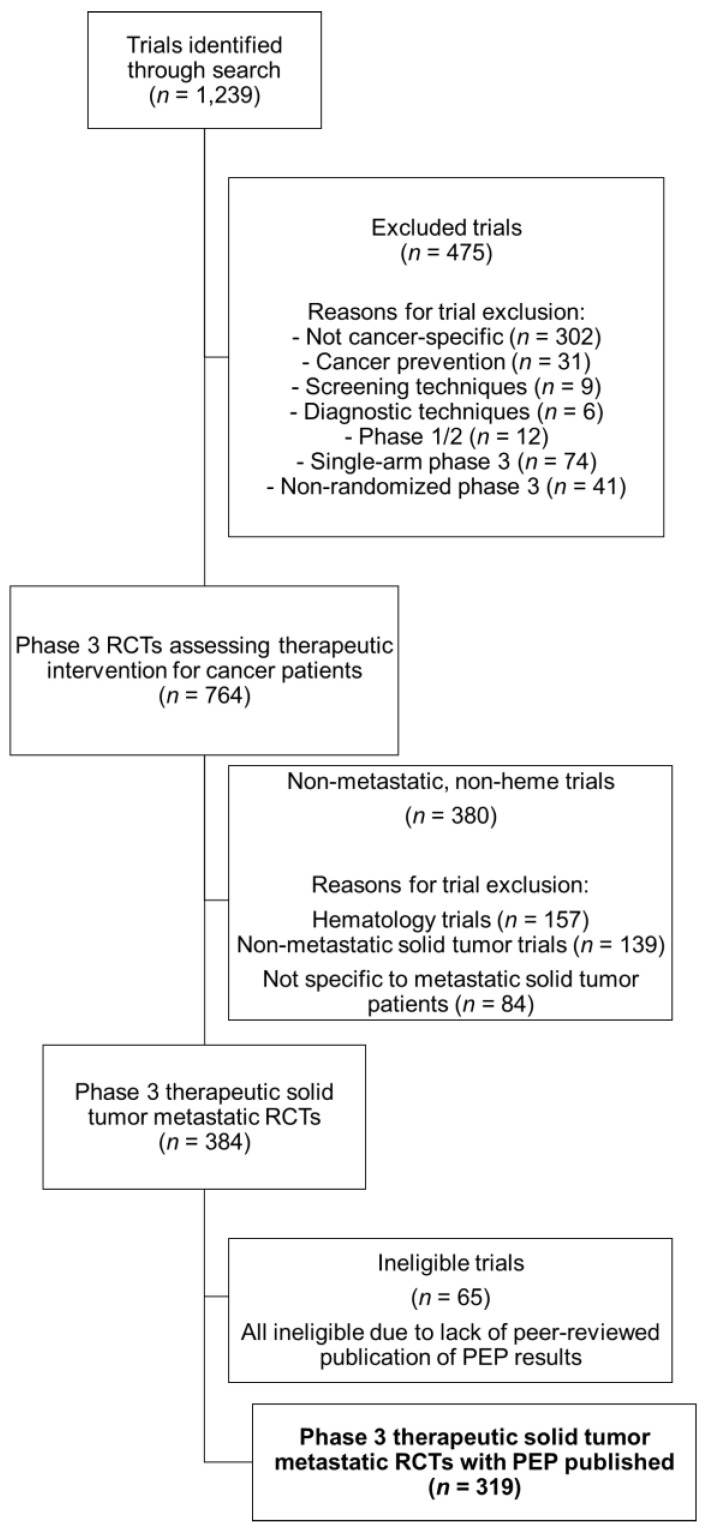
Consort diagram showing the selection criteria for phase three randomized control trials of metastatic solid tumor patients, with publications of their primary endpoints ultimately included in our analysis.

The initial trial search yielded 1239 trials, of which 764 were randomized multi-arm phase three cancer clinical trials assessing a therapeutic intervention, and 380 studies were excluded for not being specific to patients with metastatic solid tumors. Of the remaining 384 trials, 65 were ineligible because of the absence of peer-reviewed publication of primary endpoint (PEP) results. Three-hundred and nineteen phase three metastatic solid tumor clinical trials met the inclusion criteria and were analyzed (Figure 1). These studies, with a combined total enrollment of 209,366 patients, were initiated enrollment between 1995 and 2014. Of these, 287 (90%) were industry-funded and 60 (19%) were cooperative-group-sponsored (Table 1). Industry and cooperative group sponsorship were not mutually exclusive. Of the 60 identified cooperative group trials, 53% (32 trials) were also industry-supported, while just 11% of industry-funded trials were cooperative-group-sponsored.

TTR was first defined as the time from trial enrollment completion to the first PEP results. Among all 319 trials, the median TTR was 31.0 months (IQR 22.0–41.0 months). Industry-funded trials took 9.0 fewer months to report than non-industry-funded trials (31.0 vs. 40.0 months; *p* = 0.005; Table 1). Similarly, successful trials (defined as meeting the PEP) reported results 8.5 months faster than unsuccessful trials (27.5 vs. 36.0 months; *p* < 0.001). In contrast, cooperative-group trials took 6.5 months longer to report than non-cooperative group trials (37.5 vs. 31.0 months; *p* < 0.001). Trials using surrogate endpoints reported 2.5 months faster than those using non-surrogate endpoints (30.0 vs. 32.5 months; *p* = 0.03; Table 1). Trials involving second-line or subsequent-line therapies reported 5.0 months faster than those involving first-line therapies (28.0 vs. 33.0 months, *p* = 0.001). There was no difference in TTR by disease site (*p* = 0.45), treatment modality (*p* = 0.86), trial design (*p* = 0.41), or enrollment region (*p* = 0.30). Multiple regression modeling demonstrated that trial success (*p* < 0.001), subsequent-line therapy utilization (*p* = 0.04), and industry support (*p* = 0.006) were independently associated with a shorter TTR. Cooperative group status (*p* = 0.18) was not independently associated with a longer TTR, nor was surrogate endpoint utilization (*p* = 0.53).

Differential enrollment dynamics between trials may impact the post-accrual-completion time for sufficient events to report results. Therefore, to determine if the identified TTR covariates above remained consistent, irrespective of accrual dynamics, a sensitivity analysis was performed examining TTR from enrollment initiation rather than enrollment completion. Consistent with the initial analysis, industry-funded trials took 30.5 fewer months to report (58.0 vs. 88.5 months; *p* < 0.001; Table 1), cooperative group trials took 25.5 months longer to report (81.5 vs. 56.0 months; *p* < 0.001), trial success was associated with an 18.0 month reduction in TTR (52.0 vs. 70.0 months; *p* < 0.001), subsequent-line therapy was associated with a 10.0 month reduction in TTR (63.0 vs.. 53.0 months; *p* < 0.001), and use of surrogate endpoints was associated with a 9.0 month reduction in TTR (54.0 vs. 63.0 months; *p* = 0.003). Notably, multinational enrollment was associated with a shorter TTR, with trials accruing in the U.S. alone taking 77.5 months to report, compared with 58.0 months for multinational trials (*p* = 0.001). Multiple regression modeling confirmed the independent effects of industry support (*p* = <0.001), line of therapy (*p* = 0.03), and PEP success (*p* < 0.001) on TTR, as well as that of cooperative group sponsorship (*p* < 0.001). Multinational trial enrollment also remained independently associated with a faster rate of TTR (*p* = 0.003). 

## 3. Discussion

These data demonstrate the association between the TTR of clinical trials in oncology and two of the main drivers of clinical trials, namely: multi-institutional cooperative groups and industry support. This study suggests that cooperative group trials may lag behind their non-cooperative-group-supported counterparts at multiple stages of clinical trial development, including study accrual and post-trial reporting. These delays may relate in part to differences in the design or execution of industry and cooperative group trials. Concerns exist regarding the influence of industry sponsorship of clinical trials on study design and execution [9]. Industry-funded trials are associated with patient enrollment disparities in terms of age, sex, and race/ethnicity compared with non-industry-funded trials [10,11,12]. Furthermore, industry-funded trials have been associated with a higher likelihood of reporting a positive primary outcome and with the use of surrogate endpoints [13,14], which demonstrably reduces the time required to complete clinical trials [15]. Despite these concerns, our data suggest that industry-funded trials independently report findings more quickly than cooperative-group trials, irrespective of surrogate endpoint usage or trial success. 

A myriad of factors may contribute to the differences in speed between industry-funded and cooperative group trials, such as differing organizational structures or levels of administrative support. Evaluating the root causes of these differences merits further study. These differences likely have an effect on the speed of execution and reporting of clinical trials, in part owing to the known regulatory and administrative burden associated with running clinical trials. Survey data suggest that common sources of regulatory burden following trial completion include “sponsor queries of databases and access to records, sponsor-required closeout activities, and long-term follow-up” [16]. These cited reasons for post-trial delay impact both industry and cooperative group sponsored trials alike. However, cooperative groups in particular have publicly acknowledged their difficulty with effectively responding to these challenges, spurring calls in the past decade to improve the efficiency of results reporting, and to reduce superfluous regulatory and reporting requirements in cooperative-group-sponsored trials [3]. The challenges faced by cooperative groups may reflect less robust administrative funding or more burdensome internal regulatory practices amongst cooperative-group trials. In contrast, trials sponsored by industry come with a fiduciary responsibility to shareholders not present for cooperative group sponsored trials. Given the high potential rewards associated with investment in research and development for cancer drug approvals [17], amongst industry-sponsored trials there inherent financial incentives exist for rapid trial accrual and for the interpretation and dissemination of results that may not exist to the same degree or nature for their cooperative-group-sponsored counterparts. These differences in incentives may drive structural differences between industry funded and cooperative-group-sponsored trials that ultimately influence TTR.

The use of surrogate endpoints such as response rate and progression-free survival has been associated with a reduction in oncologic clinical trial reporting times [15]. Echoing these findings, our results demonstrate an association between surrogate endpoint use and reduced TTR on univariate analysis, although this association was not observed on multivariate analysis. Notably, multivariate analysis involved adjustment by industry sponsorship, so a lack of signal on multivariate analysis may reflect a link between industry sponsorship and the use of surrogate endpoints—an association which has been previously demonstrated [15]. Furthermore, line of therapy was also associated with differential clinical trial reporting times, with trials of second-line or subsequent-line therapies having a shorter TTR than trials for first-line therapies [15]. Patient cohorts in trials of second-line therapies may have more advanced disease characteristics and higher event rates, shortening follow-up periods and lowering the sample sizes required for adequate statistical power, which may shorten study accrual times.

Our data also suggest that multinational phase three trials are associated with more timely trial enrollment and reporting. Multinational trials may benefit from shorter enrollment periods owing to a lower average number of patients enrolled per study site [18]. It is possible that these upfront time-savings may outweigh pre-accrual delays associated with the initiation of a multinational trial, such as additional regulatory hurdles associated with multinational rather than uninational enrollment initiation. These data highlight a further potential benefit of increasing multinational clinical trials, which have, in other studies, demonstrated higher completion rates than trials performed exclusively in the United States [19].

TTR, as defined from enrollment initiation, may be impacted by factors that are not accounted for in this study, such as trial amendments during study accrual. Trial amendments can be related to a variety of factors, such as the publication of new randomized data, changes to the standard(s) of care during trial accrual, or modification (specifically broadening) of trial eligibility criteria in response to slow accrual. Regardless of the cause, amendments to trial protocols may be associated with TTR delays, owing to a mandatory review of protocol amendments by both regulatory agencies and the study sponsor(s). Additionally, the particular design of the trial enrollment can also affect TTR. For instance, a trial with a higher sensitivity for halting accrual during interim analyses upon detection of a significant difference between treatment arms (or a trial with more frequent interim analyses) may be more likely to close earlier, thereby impacting TTR.

Trial success was independently associated with a shorter TTR in this analysis. There are several reasons trials with positive results may report faster than those with negative results, including academic recognition or financial incentives. However, the timely dissemination of negative results is also of importance for the advancement of the field of oncology. Negative trial results may prompt changes to clinical practice patterns in the same way positive results do. Furthermore, negative trial results can inform modifications to the protocols of active clinical trials. Delays in the publication of negative resulting in absent financial incentives may be driven by the need to pursue additional sub-analyses of trial data, sometimes unplanned, in an attempt to mine further noteworthy, publishable conclusions. Regardless of the reason, the clinical and research implications of trial data should prompt a careful examination of solutions to mitigate delays in the reporting of negative trial results.

This study has certain limitations. The use of enrollment completion as time zero presumes that study duration after enrollment completion is not materially different among studies spanning multiple disease sites. To mitigate this limitation, we focused only on trials of metastatic solid tumors, whose disease dynamics and overall survival are, broadly-speaking, similar across tumor sites compared with their non-metastatic trial counterparts. We acknowledge that this choice necessarily limited our sample of phase three trials in oncology, and any differences between the clinical trials of metastatic and non-metastatic solid tumors was not assessed by this study. To further mitigate the effects of heterogeneity and sample variability in trials, we included disease site as a covariate in the univariate analysis of TTR, as well as line of therapy; notably, line of therapy, but not disease site, was independently associated with TTR. Finally, the study’s sensitivity analysis examining TTR from enrollment initiation rather than enrollment completion corroborated the main findings of the original analysis, and serves as an additional control for heterogeneity in accrual dynamics across trials. Another potential confounding factor includes the use of a publication date as a time marker for time to reporting. Date of publication rather than date of acceptance was used in this analysis, given the inconsistent availability of manuscript acceptance dates in the literature. Journals have variable time lags between manuscript acceptance to eventual publication that relate to internal practices, as well as the particular objectives of a journal at any given point in time; manuscripts that have been through multiple journal submission processes (that is, rejected from one journal and submitted to another) may similarly experience publication-related delays in the TTR window. This variability in publication-specific considerations may influence TTR. Additionally, this analysis included only trials with published outcomes, likely excluding trials less likely to report positive findings. Nevertheless, this study shows that cooperative group trials are independently associated with longer TTR, independent of trial success. In order to combat publication bias in future studies of TTR dynamics, prompt and compliant reporting of results to ClinicalTrials.gov is encouraged [20].

## 4. Materials and Methods

To identify relevant trials, ClinicalTrials.gov was queried for phase three clinical oncology trials for patients with metastatic solid tumors. Metastatic solid tumor trials were chosen to reduce heterogeneity in disease dynamics across disease sites, which could affect TTR. ClinicalTrials.gov was queried using the following parameters: terms, “cancer”; study type, “all studies”; status, excluded “not yet recruiting”; phase, phase three; and study results, “with results.” From this search, trials specific to patients with metastatic solid tumors with the publication of primary endpoint (PEP) results were identified. TTR was defined from either enrollment completion or enrollment initiation, as specified below, to time of peer-reviewed publication of PEP results. PubMed.gov was searched to identify peer-reviewed publications containing trial PEP results and publication dates. Univariate analyses of TTR with respect to industry funding, cooperative group sponsorship, trial success, treatment modality, line of therapy (first-line vs. subsequent lines), disease site, enrollment region, primary endpoint (surrogate vs. non-surrogate), and study design (non-inferiority vs. superiority) were performed using Mann–Whitney U and Kruskal–Wallis tests (Mann–Whitney U-tests for two-sample comparisons, and Kruskal–Wallis tests for comparisons of three or more samples). Multiple regression modeling was performed using covariates with a signal identified (*p* < 0.05) on the univariate analysis. The covariates that were ultimately included in the multivariate analysis using TTR from enrollment completion were industry funding, cooperative group sponsorship, trial success, line of therapy, and primary endpoint; multivariate analysis using TTR from enrollment initiation additionally included the enrollment region. All of the statistical analyses were performed using the IBM SPSS Statistics software package (Version 22.0, IBM Corp, Armonk, NY, USA). 

## 5. Conclusions

In summary, time to reporting of clinical trials in oncology is associated with both industry support and cooperative group sponsorship, with a shorter time to reporting amongst industry-funded trials than cooperative-group trials. Our study highlights an opportunity for cooperative group trials to focus efforts on reducing post-enrollment reporting time. 

## Figures and Tables

**Table 1:** Factors associated with time to reporting (TTR) from enrollment completion and initiation. Univariate and multiple regression model.

Factor		No. (%)	Median TTR from Enrollment Completion in Months	Univariate *p*-Value *	Multiple Regression *p*-Value	Median TTR from Enrollment Initiation in Months	Univariate *p*-Value *	Multiple Regression *p*-Value
Overall		319	31.0 (IQR: 22.0–41.0)	-		60.0 (IQR: 47.0–77.0)	-	
Cooperative-group-sponsorship	No	259 (81.2)	31.0	<0.001	0.18	56.0	<0.001	0.001
	Yes	60 (18.8)	37.5			81.5		
Industry sponsorship	No	32 (10.0)	40.0	0.005	0.006	88.5	<0.001	<0.001
	Yes	287 (90.0)	31.0			58.0		
Trial success ^‖^	No	143 (44.8)	36.0	<0.001	<0.001	70.0	<0.001	<0.001
	Yes	176 (55.2)	27.5			52.0		
Treatment modality ^‡^	Chemotherapy	167 (52.4)	31.0	0.86		60.0	0.09	
	Targeted therapy	110 (34.5)	32.0			59.0		
	Supportive care	40 (12.5)	29.0			64.0		
	Radiation therapy	2 (0.6)	25.0			119.5		
Line of therapy	First-line	155 (55.2)	33.0	0.001	0.04	63.0	<0.001	0.03
	Second-line or later	126 (44.8)	28.0			53.0		
Disease site	Breast	53 (16.6)	35.0	0.45		68.0	0.20	
	Genitourinary	44 (13.8)	26.5			59.0		
	Gastrointestinal	64 (20.1)	31.5			58.5		
	Thoracic	77 (24.1)	31.0			55.0		
	Other ^§^	81 (25.4)	31.0			63.0		
Enrollment region	Multinational	260 (81.5)	31.0	0.30		58.0	0.001	0.003
	USA-only	38 (11.9)	32.0			77.5		
	Non-USA single country	21 (6.6)	33.0			69.0		
Primary endpoint ^^^	Nonsurrogate	124 (44.4)	32.5	0.03	0.53	63.0	0.003	0.12
	Surrogate	155 (55.6)	30.0			54.0		
Study design	Noninferiority	262 (86.7)	31.0	0.41		59.5	0.07	
	Superiority	37 (12.4)	32.0			65.0		

* Univariate analysis performed with Mann–Whitney U-tests (by cooperative-group-sponsorship, industry-sponsorship, trial success, primary endpoint, line of therapy, and study design) and Kruskal–Wallis tests (by treatment modality, disease site, and enrollment region). ^‖^ Trial success refers to trials where the primary endpoint was met. ^‡^ Treatment modality refers to the primary intervention as part of trial randomization. Chemotherapy generally includes cytotoxic agents, whereas targeted therapy includes monoclonal antibodies, small-molecule inhibitors, and similar. ^§^ Other disease site trials include single-disease site trials of head and neck, gynecologic, and skin malignancies, as well as trials allowing multiple disease sites. ^^^ Primary endpoint divided into nonsurrogate and surrogate endpoints; nonsurrogate endpoints include direct measures of quantity or quality of life (such as overall survival), or direct disease-related outcomes (such as disease-free survival). Surrogate endpoints include radiographic or pathologic endpoints (primarily progression-free survival and pathology or radiographic response rates).

## References

[1] Doroshow J.H. (2013). Timely completion of scientifically rigorous cancer clinical trials: An unfulfilled priority. J. Clin. Oncol..

[2] Dilts D.M., Sandler A.B., Cheng S.K., Crites J.S., Ferranti L.B., Wu A.Y., Finnigan S., Friedman S., Mooney M., Abrams J. (2009). Steps and time to process clinical trials at the Cancer Therapy Evaluation Program. J. Clin. Oncol..

[3] Nass S., Moses H., Mendelsohn J., Institute of Medicine (US) Committee on Cancer Clinical Trials and the NCI Cooperative Group Program (2010). A National Cancer Clinical Trials System for the 21st Century: Reinvigorating the NCI Cooperative Group Program.

[4] Ehrhardt S., Appel L.J., Meinert C.L. (2015). Trends in National Institutes of Health Fundingfor Clinical Trials Registered in ClinicalTrials.gov. JAMA.

[5] Abrams J.S., Mooney M.M., Zwiebel J.A., Korn E.L., Friedman S.H., Finnigan S.R., Schettino P.R., Denicoff A.M., Kruhm M.G., Montello M. (2013). Implementation of timeline reforms speeds initiation of National Cancer Institute-sponsored trials. J. Natl. Cancer Inst..

[6] Dilts D.M., Sandler A.B. (2006). Invisible Barriers to Clinical Trials: The Impact of Structural, Infrastructural, and Procedural Barriers to Opening Oncology Clinical Trials. J. Clin. Oncol..

[7] Williams E., Brown T.J., Griffith P., Rahimi A., Oilepo R., Hammers H., Laetsch T.W., Currykosky P., Partridge S., Beg M.S. (2020). Improving the Time to Activation of New Clinical Trials at a National Cancer Institute–Designated Comprehensive Cancer Center. JCO Oncol. Pract..

[8] Hoos W.A., James P.M., Rahib L., Talley A.W., Fleshman J.M., Matrisian L.M. (2013). Pancreatic Cancer Clinical Trials and Accrual in the United States. J. Clin. Oncol..

[9] Lexchin J., Bero L.A., Djulbegovic B., Clark O. (2003). Pharmaceutical industry sponsorship and research outcome and quality: Systematic review. BMJ.

[10] Murthy V.H., Krumholz H.M., Gross C.P. (2004). Participation in cancer clinical trials: Race-, sex-, and age-based disparities. JAMA.

[11] Ludmir E.B., Mainwaring W., Lin T.A., Miller A.B., Jethanandani A., Espinoza A.F., Mandel J.J., Lin S.H., Smith B.D., Smith G.L. (2019). Factors associated with age disparities among cancer clinical trial participants. JAMA Oncol..

[12] Grant S.R., Lin T.A., Miller A.B., Mainwaring W., Espinoza A.F., Jethanandani A., Walker G.V., Smith B.D., Ashleigh Guadagnolo B., Jagsi R. (2020). Racial and Ethnic Disparities among Participants in US-Based Phase 3 Randomized Cancer Clinical Trials. JNCI Cancer Spectr..

[13] Roper N., Zhang N., Korenstein D. (2014). Industry collaboration and randomized clinical trial design and outcomes. JAMA Intern. Med..

[14] Pasalic D., McGinnis G.J., Fuller C.D., Grossberg A.J., Verma V., Mainwaring W., Miller A.B., Lin T.A., Jethanandani A., Espinoza A.F. (2020). Progression-free survival is a suboptimal predictor for overall survival among metastatic solid tumour clinical trials. Eur. J. Cancer.

[15] Chen E.Y., Joshi S.K., Tran A., Prasad V. (2019). Estimation of Study Time Reduction Using Surrogate End Points Rather Than Overall Survival in Oncology Clinical Trials. JAMA Intern. Med..

[16] Vose J.M., Levit L.A., Hurley P., Lee C., Thompson M.A., Stewart T., Hofacker J., Bruinooge S.S., Hayes D.F. (2016). Addressing Administrative and Regulatory Burden in Cancer Clinical Trials: Summary of a Stakeholder Survey and Workshop Hosted by the American Society of Clinical Oncology and the Association of American Cancer Institutes. J. Clin. Oncol..

[17] Tay-Teo K., Ilbawi A., Hill S.R. (2019). Comparison of Sales Income and Research and Development Costs for FDA-Approved Cancer Drugs Sold by Originator Drug Companies. JAMA Netw. Open.

[18] Yusuf S., Wittes J. (2016). Interpreting Geographic Variations in Results of Randomized, Controlled Trials. N. Engl. J. Med..

[19] Stensland K.D., McBride R.B., Latif A., Wisnivesky J., Hendricks R., Roper N., Boffetta P., Hall S.J., Oh W.K., Galsky M.D. (2014). Adult Cancer Clinical Trials That Fail to Complete: An Epidemic?. J. Natl. Cancer Inst..

[20] DeVito N.J., Bacon S., Goldacre B. (2020). Compliance with legal requirement to report clinical trial results on ClinicalTrials.gov: A cohort study. Lancet.

